# Chronic Nicotine Exposure Abolishes Maternal Systemic and Renal Adaptations to Pregnancy in Rats

**DOI:** 10.1371/journal.pone.0150096

**Published:** 2016-02-25

**Authors:** Vanessa Meira Ferreira, Clevia Santos Passos, Edgar Maquigussa, Roberto Braz Pontes, Cassia Toledo Bergamaschi, Ruy Ribeiro Campos, Mirian Aparecida Boim

**Affiliations:** 1 Renal Division, Department of Medicine, Federal University of São Paulo, São Paulo, Brazil; 2 Cardiovascular Division, Department of Physiology, Federal University of São Paulo, São Paulo, Brazil; University Medical Center Utrecht, NETHERLANDS

## Abstract

Pregnancy is characterized by maternal systemic and intrarenal vasodilation, leading to increases in the renal plasma flow (RPF) and glomerular filtration rate (GFR). These responses are mainly mediated by nitric oxide (NO) and relaxin. The impact of cigarette smoking on the maternal adaptations to pregnancy is unclear. Here we evaluated the effects of chronic exposure to nicotine on systemic and intrarenal parameters in virgin (V) and 14-day pregnant (P) Wistar rats. V and P groups received saline or nicotine (6 mg·kg^-1^·day^-1^) respectively, via osmotic minipumps for 28 days, starting 14 days before pregnancy induction. Nicotine induced a 10% increase in blood pressure in the V group and minimized the characteristic pregnancy-induced hypotension. Renal sympathetic nerve activity (rSNA) and baroreflex sensitivity were impaired by nicotine mainly in the P group, indicating that the effect of nicotine on blood pressure was not mediated by nervous system stimulation. Nicotine had no effect on GFR in the V rats but reduced GFR of the P group by 30%. Renal expression of sodium and water transporters was downregulated by nicotine, resulting in increased fractional sodium excretion mainly in the P group, suggesting that nicotine compromised the sodium and water retention required for normal gestation. There was a reduction in the expression of inducible NO synthase (iNOS) in both the kidney tissue and renal artery, as well as in the expression of the relaxin receptor (LGR7). These results clearly show that nicotine induced deleterious effects in both virgin and pregnant animals, and abolished the maternal capacity to adapt to pregnancy.

## Introduction

A normal pregnancy is characterized by maternal adaptations that include an increase in cardiac output with peripheral and intrarenal vasodilation. In addition, these adaptations are followed by an 80 and 50% rise in the renal plasma flow (RPF) and glomerular filtration rate (GFR), respectively [1]. Relaxin, estrogen, prostaglandins and nitric oxide (NO) modulate these adaptations [2–5]. Pregnancy also imposes a necessary expansion of the extracellular volume (ECV) by sodium and water retention via mechanisms involving changes in the expression of tubular transporters. We recently demonstrated that the expression of renal transporters including the Na-Cl and Na-K-2Cl cotransporters, Na-H exchanger, and aquaporin 2 is increased during pregnancy in rats [6, 7].

Despite the increasing number of antismoking campaigns, the World Health Organization (WHO) currently estimates that smoking accounts for about 6 million deaths worldwide each year [8]. Nicotine is an alkaloid that is systemically absorbed and subsequently distributed to several organs including the kidney, which is responsible for 30% of its metabolism. In humans, 80% of nicotine is converted into cotinine by two main enzyme mechanisms, which are the cytochrome P450 (CYP) and cytosolic aldehyde oxidase [9, 10]. In the CYP pathway, nicotine is metabolized to cotinine by the enzyme CYP2A6 primarily in the liver. In the rat kidney, the main isoform responsible for nicotine metabolism is the CYP1A1/2 [9, 11, 12]. Nicotine metabolism is influenced by numerous factors such as age, sex, and genetic background, as well as pregnancy, which increases its metabolism [9, 10, 12–15].

The effects of nicotine on renal function are not clear. Acute nicotine administration resulted in an increase in renal vascular resistance, with a reduction in RPF and GFR [16–18]. Moreover, chronic smokers were shown to develop microalbuminuria with a rapid progression to proteinuria, due to thickening of the glomerular basement membrane and endothelial cell activation [16, 19]. On the other hand, other studies suggest that nicotine-induced proteinuria has a tubular origin since albuminuria has not been detected [20]. In addition, it has been suggested that chronic exposure to nicotine induces tolerance, which attenuates these effects [14, 16].

Nicotine and cotinine cross the fetoplacental barrier and are concentrated in the amniotic fluid, umbilical cord, and fetal circulation [21–23], reaching levels up to 10-fold higher than in the maternal circulation [16, 24]. Although the deleterious effects of nicotine on fetal development have been extensively investigated, few studies have focused on the maternal organism, especially on relevant maternal adaptations to pregnancy. Therefore, this study evaluated the effects of chronic nicotine exposure before and during pregnancy on the maternal systemic circulation, sympathetic vasomotor modulation of the kidneys, and renal function. We discovered that nicotine abolished the systemic and intrarenal adaptations to pregnancy. Furthermore, the reduction in the expression of relaxin receptors and inducible (i) NOS in the kidney may be the main factors responsible for these effects.

## Methods

### Experimental Protocol

Adult virgin female Wistar rats (200–250 g) were obtained from the Animal Care Facility (CEDEME) of the Federal University of São Paulo, and the experimental protocol was approved by and followed the guidelines of the Ethical Committee of the Federal University of São Paulo (1818/2009). The animals had free access to standard rat chow and tap water and were maintained in a temperature-controlled environment (23°C) on a 12-h light/dark cycle. The rats were pair-housed with an adult male Wistar rat for 2–3 days with daily vaginal examinations up to the day sperm was first detected in the vaginal smears, which was considered pregnancy day 1.

Saline or nicotine (6 mg·kg^-1^·day^-1^) was administered via subcutaneously implanted osmotic minipumps (2ML4 model, Alzet Pumps, Cupertino, CA, USA), as previously described [25]. This daily dose is similar to the amount of nicotine contained in approximately 15 cigarettes [26]. The animals were allocated to four groups and treated for 28 days as follows: virgin rats, treated with saline (V) or nicotine (VN) and 14-day pregnant rats treated with saline (P) or nicotine (PN). In the pregnant group, nicotine administration started 14 days before pregnancy induction and continued during 14 days of pregnancy when the animals were euthanized and systemic and renal parameters were evaluated. This period of pregnancy was used since it is characterized by more pronounced changes in the renal function in rats with highest levels if GFR and RPF [27].

After treatment, the rats were anesthetized with ketamine and xylazine (40 and 20 mg/kg, respectively) and their femoral arteries were catheterized for mean arterial pressure (MAP) recording using direct registration with the Power Lab system (ADInstruments, New South Wales, Australia). Renal sympathetic nervous activity (rSNA) was also measured in the same animals.

In addition, 24-h urine collection was performed using metabolic cages in another set of animals (n = 6 per group). After urine collection, the rats were anesthetized with ketamine and xylazine (40 and 20 mg/kg, respectively) for blood sampling while the kidney and renal arteries were removed and immediately frozen at -80°C until used to estimate the mRNA and protein expression levels and for immunostaining experiments. Plasma and urinary concentrations of cotinine were determined using a specific kit (BQ096D, BQKits Inc. San Diego, California, USA) following the manufacturer’s instructions. Creatinine and urinary protein concentration were measured using specific kits (Labtest and Sensiprot, Labtest Diagnostica, Lagoa Santa, MG, Brazil). Plasma and urinary sodium and potassium were estimated using a flame photometer (B462 Micronal, São Paulo, SP, Brazil).

### Measurement of MAP and rSNA

Left femoral artery was catheterized under ketamine (40 mg/kg) and xilazine (20 mg/Kg) anesthesia. Animals were allowed to recover and 24 hr after catheterization, blood pressure was assessed in conscious rats. After BP measurements and a period of stabilization, the rats were slowly anesthetized with urethane (1.4 g.kg^-1^, iv), which is known to preserve the rSNA and blood pressure basal values [28] that did not differ from those obtained under other anesthetic such as barbital sodium [29]. The mean arterial blood pressure (MAP) and heart rate (HR) were then obtained from the pulsatile blood pressure and were “on line” recorded using a PowerLab equipment (ADInstruments, Australia). A left retroperitoneal incision was made to expose the renal sympathetic nerve, which was carefully dissected and separated from the renal artery and vein and other adjacent tissues. After dissection, the renal nerve was placed on a platinum electrode in a bipolar configuration, immersed in mineral oil throughout the experiment. The renal nerve signal was visualized with the aid of an oscilloscope (TDS 220, Tektronix Inc. Beaverton, OR, USA), and the noise of the activity was assessed using an audio amplifier. The nerve activity was amplified 20.000X (Neurolog, Welwyn Garden City, UK) using a filter with a frequency range 100–1000 Hz. During the experiments, the SNA was rectified and integrated online while the neural activity was analyzed offline using the appropriate software spike histogram (ADInstruments). The responses of the rSNA to various stimuli were expressed as the percentage of change compared with the basal value obtained immediately before each test. For this purpose, the raw nerve signal was passed through a spike discriminator (PowerLab, ADInstruments) to remove background noise, and the total nerve activity was expressed as spikes/s, computed from the time it changed from the basal value (spikes/s over a 60-s period) to when it returned to basal level. Only experiments in which the level of background noise was confirmed at the end of the experiments following hexamethonium (30 mg/Kg, IV, Sigma) are included in this report.

All recordings were acquired at a sampling frequency of 2 kHz. At the end of the experiment, the rats were administered 5% potassium chloride (KCl) and considered dead after a cardiac arrest was observed.

### Analysis of the Baroreceptor Control

The control of the rSNA by arterial baroreceptors was verified by analyzing changes in the activities of renal sympathetic nerve in response to MAP variations induced by vasoactive drugs. For the renal baroreflex analysis, phenylephrine (100pg/ml) and nitroprusside (200 pg/ml) were infused at a rate of 0.2 ml/min and 0.1 ml/min respectively, with 20–25 min intervals between drugs. The arterial baroreceptor-mediated control was estimated by plotting the reflected variations of rSNA(spikes/s) in response to increases or decreases in the MAP, and was expressed as pps/mmHg [30]. Furthermore, linear regression curves were constructed from these data, and the slope of these lines was calculated, as previously reported [31].

### mRNA expression levels

The mRNA expression levels were estimated using quantitative real-time polymerase chain reaction (qPCR) using a Gene-Amp 5700 System (Applied Biosystems, USA). Total RNA was purified from the renal cortex and renal artery tissue using the phenol and guanidine isothiocyanate-cesium chloride method (Trizol, Life Technologies, Carlsbad, CA, USA). The cDNA was synthetized as previously described [6] in the presence of DNAse (Promega, Madison, WI, USA) to exclude genomic DNA contamination. The primers were designed and chosen based on their efficiency using a proper software (Primers express, Applied Biosystems, USA). The following primers were used: β-actin (5'-cctctatgccaacacagtgc-3' and 5'-acatctgctggaaggtggac-3'), Na^+^/K^+^/2Cl^-^ cotransporter (BSC, 5'-agaaacggtgttcgggcctc-3' and 5'-tctgtcatcctaagtggacactg-3'), Na^+^/H^+^ exchanger (NHE3, (5'-ttggggtacttccaaggcag-3' and 5'-agagtgtcaaagggttccac-3'), epithelial sodium channel (ENaC, 5'-cttacgggttgaacaccacca-3' and 5'-ttgcagaaccacagagcctcta-3'), renal potassium (K^+^) channel (ROMK_2_, 5'-acaggactttgagctggtggtctt-3' and 5'-ttcccttccttggtcttggacaca-3'); aquaporin (AQP) 1 (5'-actcgcttggccgcaatgac3-' and 5'-gctgagccaccaaggtcacg-3'), AQP2 (5'-ccacgctcctttttgtcttc-3' and 5'-gtccccacggattcctact-3'), relaxin receptor 7 (LGR7, 5'-tgggctcattggccgttctg-3' and 5'-actccattcgtgccgtagtag-3'), extracellular (e) NOS (5'-tcactgtagctgtgctggcataca-3' and 5'-gcaagttaggatcagctggca-3'), iNOS (5'-aggtgttcagcgtgctccag-3' and 5'-agttcagcttggcggccacc-3'), and the nicotine metabolizing enzyme, CYP1A1 (5'-tggccacttcgaccctttcaagta-3' and 5'-tgactatgctgagcagctcttggt-3'). A tube containing water instead cDNA was used as negative control.

The PCR product accumulation was monitored using SYBR Green I intercalating dye (Molecular Probes, Eugene, OR, USA), which exhibits higher fluorescence following its binding with double stranded DNA. The mRNA expression levels were normalized to β-actin expression, and the results are expressed in arbitrary units.

### Immunohistochemistry

The rat kidneys were embedded in paraffin, cut into 4-mm slices, and mounted on silanized slides. The sections were then deparaffinized and rehydrated with decreasing concentrations of alcohol. Antigen retrieval was performed with Tris- ethylenediaminetetraacetic acid (EDTA) buffer pH 9.0 (10 mM Tris and 1 mM EDTA, Sigma-Aldrich Corp., St. Louis, MO, USA) while the endogenous peroxidase was blocked with 3% hydrogen peroxide (Merck, Brazil). Then, the slices were incubated with a blocking protein (Dako, USA) to avoid unspecific bindings. The sections were then incubated with the relaxin receptor (LGR7) antibody (goat polyclonal, 1:100 dilution, Santa Cruz Biotechnology, Santa Cruz, CA, USA) for 12 h at 4°C. After washing in phosphate-buffered saline (PBS), the slides were incubated with horseradish peroxidase (HRP)-polymer conjugate (Dako, Real Carpinteria, CA, USA). The bound antibody was visualized using diaminobenzidine (DAB) dye (Dako, Real Carpinteria, CA, USA) and counterstained with hematoxylin (Merck, Taquara, RJ, Brazil). The analysis was performed using a light microscope (Leica Imaging Systems) and the stained proteins were quantified using the Corel Photo-Paint program and a quantitative analysis software developed at the University of Texas (Image Tool, UTHSCSA).

### Renal Function

The GFR was evaluated in conscious rats using a fluorescent-labeled inulin clearance method with fluorescein isothiocyanate (FITC-inulin, Sigma-Aldrich Corp., St. Louis, MO, USA), as previously described [32]. In brief, FITC-labeled inulin was diluted with 1.5% saline and protected from light. To remove the residual free FITC, the solution was dialyzed using a 1000 Da cut-off dialysis membrane (Spectra/Por® 7 Membrane WR Research, USA). The animals were anesthetized with ketamine and xylazine (40 and 20 mg.kg^-1^, respectively), and their femoral veins were catheterized with a PE10 catheter connected to a PE50 catheter, which was subcutaneously tunneled out to the dorsum, exteriorized near the shoulder girdle, fixed, and then closed with metal pins. A period of 24 h was allowed to elapse to blunt possible unwanted effects of the anesthetic during the experiment. Approximately 1 mL of the FITC-inulin solution was infused via the femoral vein, and then blood samples were collected via a small cut at the tip of the tail in pre-established intervals (1, 3, 7, 10, 15, 25, 35, 55, 75, 95, 125, and 155 min). The samples were immediately transferred to a dark microplate containing HEPES buffer (500 mM, pH 7.5) and the fluorescence was measured at 496 and 520 nm excitation and emission wavelengths, respectively using a plate reader (Spectra Max, Molecular Devices, Sunnyvale, CA, USA). The inulin clearance rate was calculated using data analysis as described by Gabrielsson and Weiner [33].

### Statistical Analysis

The results are presented as the mean ± standard error (SE) and the data were evaluated using a two-way analysis of variance (ANOVA) followed by the Tukey’s post hoc test. When appropriated, the Student *t* test was used to compare the effect of pregnancy independently of the nicotine exposure. Statistical significance was defined as p<0.05.

## Results

### Nicotine Metabolism

**Fig 1:**
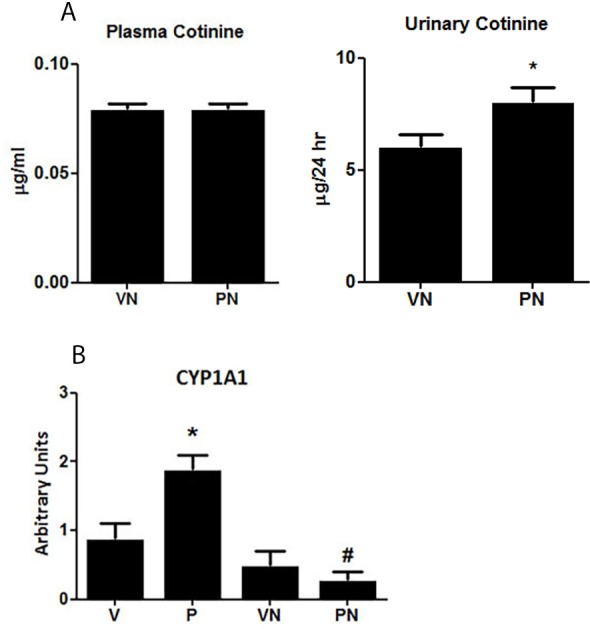
(**A**) Cotinine levels (μg/mL) in plasma and urine (n = 6group). (**B**) mRNA expression levels of cytochrome P450 (CYP) 1A1 enzyme in renal cortex (n = 8/group); *p<0.05 and ^#^p<0.05 vs virgin nicotine-treated (VN) and pregnant (P) groups, respectively.

The presence of cotinine in animals treated with nicotine indicated the efficient release of nicotine through the osmotic minipumps. The plasma cotinine concentration was similar between virgin and pregnant rats; however, urinary excretion of cotinine was higher in pregnant animals (Fig 1A). The CYP1A1 enzyme was constitutively expressed in the kidneys, and the mRNA levels significantly increased in the kidneys of the untreated (control) P rats compared to the V group (Fig 1B). This observation suggests that this enzyme may play a role in modulating the renal metabolic rate during gestation. Interestingly, chronic exposure to nicotine completely abrogated this increase in the PN group.

### Cardiovascular and Autonomic Function

**Fig 2:**
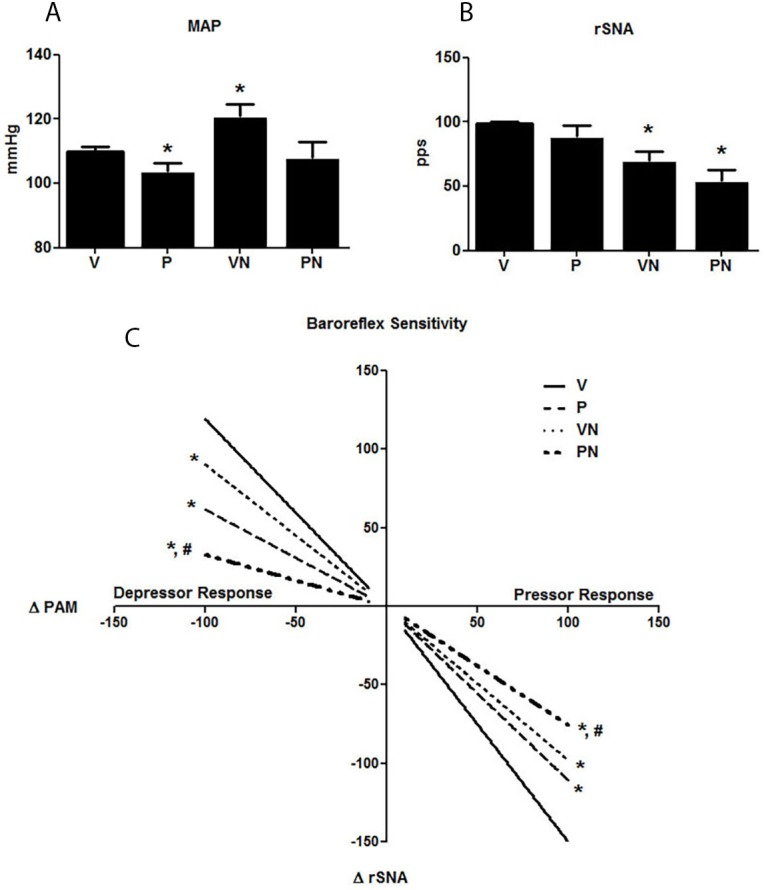
Systemic parameters (**A**) Mean Arterial Pressure (MAP), comparison between Pregnant and Virgin groups was done by using the Student *t* test. **(B)** Direct registration of renal sympathetic nerve activity (rSNA) expressed in spikes/s. (**C**) Baroreceptor reflex of rSNA in response to decrease MAP induced by acute administration of sodium nitroprusside (5, 15, and 20 mg.kg^-1^, depressor responses) and increased MAP induced by acute administration of phenylephrine (3, 5, and 10 mg.kg^-1^, pressor responses, ∆MAP/rSNA); n = 7/group, *p<0.05 and ^#^p<0.05 vs virgin (V) and pregnant (P) groups, respectively.

Fig 2A shows that pregnant rats presented lower levels of MAP compared to the virgin rats, according to the expected hypotensive effect induced by pregnancy. Nicotine induced a significant increase in the MAP in virgin but not in the pregnant rats; however, the decline in MAP, which is typical of gestation, was minimized by nicotine since the MAP of PN group was statistically similar to V group. Heart rate was unchanged among groups (data not shown). The P group animals presented no significant alterations in the rSNA compared with those in the V group. In contrast, the baroreflex sensitivity was lower in rats in the P group than it was in those in the V group. Unexpectedly, nicotine reduced the rSNA in both PN and VN group rats (Fig 2B), and this reduction was followed by a decreased in baroreflex sensitivity for rSNA control in both nicotine-treated groups (Fig 2C).

### Glomerular and Tubular Function

**Fig 3:**
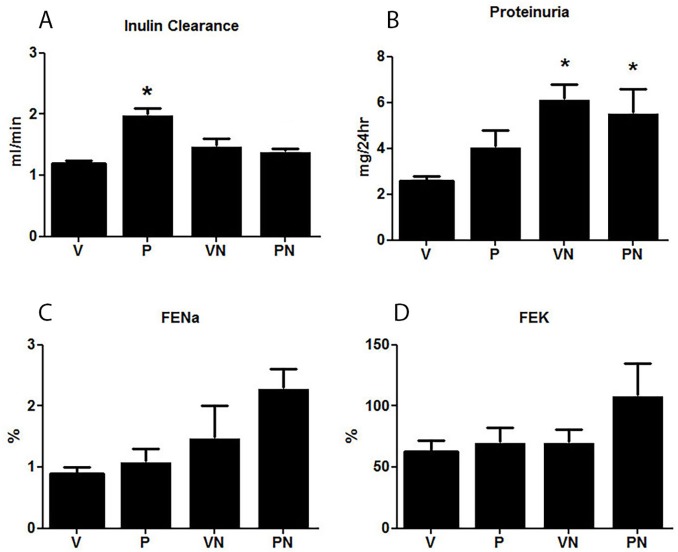
(**A**) Creatinine clearance estimated by fluorescein isothiocyanate (FITC)-labeled inulin method (mL/min) (n = 7/group); (**B**) Proteinuria mg/24 h. (**C**) Fractional sodium excretion (FENa %) (**D**) Fractional potassium excretion (FEK %); n = 6/group, *p<0.05 vs virgin (V) group.

The GFR was increased in the P group of rats, which reflected the typical maternal response to pregnancy (Fig 3A). Nicotine had no effect on GFR level of the VN group, but the hyperfiltration induced by pregnancy was minimized by the nicotine exposure. GFR in PN group was similar to V group. Nicotine induced a slight but significant increase in proteinuria in both groups (Fig 3B). Despite exhibiting a higher GFR than the other groups, the fractional excretion of sodium and potassium was not changed in the control P group (Fig 3C and 3D), indicating a higher tubular reabsorption. Nicotine did not alter fractional excretions of Na and K in the VN and in PN groups, in spite of a tendency of both FENa and FEK to increase in the PN group.

**Fig 4:**
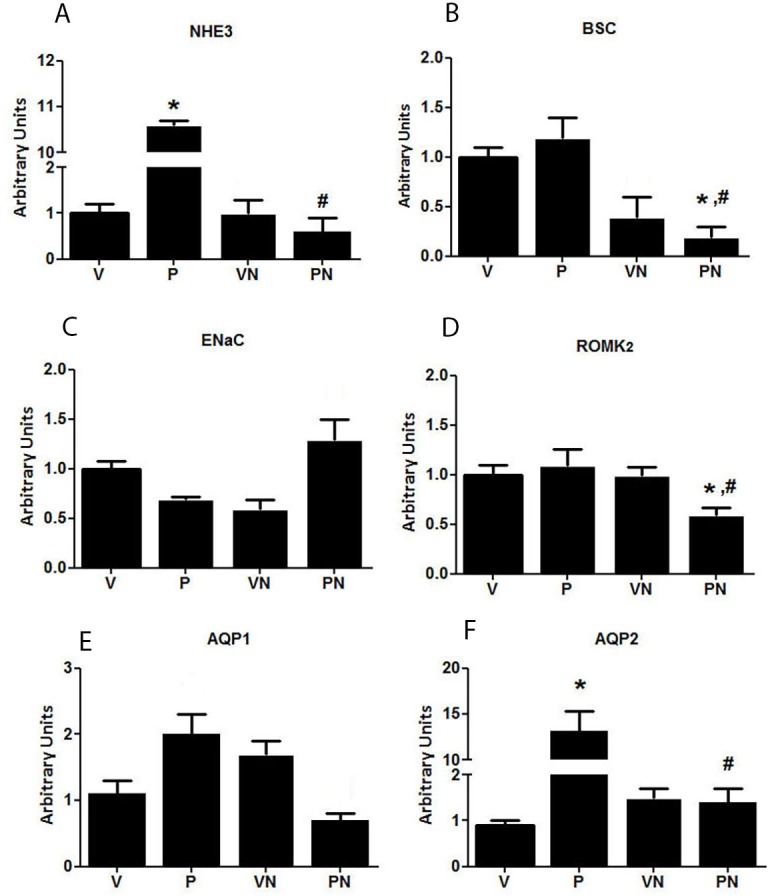
mRNA expression levels of tubular transporters (**A**) Na^+^/H^+^ exchanger (NHE_3_), (**B**) Na^+^/K^+^/2Cl^-^cotransporter (BSC), (**C**) epithelial sodium channel (ENaC), (**D**) potassium channel (ROMK_2_), (**E**). Aquaporin (AQP) 1, and (**F**) AQP2; n = 8/group, *p<0.05 and ^#^p<0.05 vs virgin (V) and pregnant (P) groups, respectively.

The increased sodium reabsorption observed in the P group rats was likely caused by the rise in the mRNA expression levels of NHE3, as shown in Fig 4. The mRNA levels of the distal sodium transporter, BSC, were not altered by pregnancy or nicotine. However, pregnant animals that were administered nicotine displayed an impressive reduction mainly in the NHE3 but also in the BSC mRNA expressions. There was a discrete but significant reduction in the K channel isoform ROMK_2_ in the PN group. The levels of AQP1 were not significantly altered among groups, however there was an impressive increase in the AQP2 levels in the control P group (Fig 4). Furthermore, nicotine significantly decreased the expression of AQP2 to levels near the control V group.

### Vasodilator Hormone Activity

**Fig 5:**
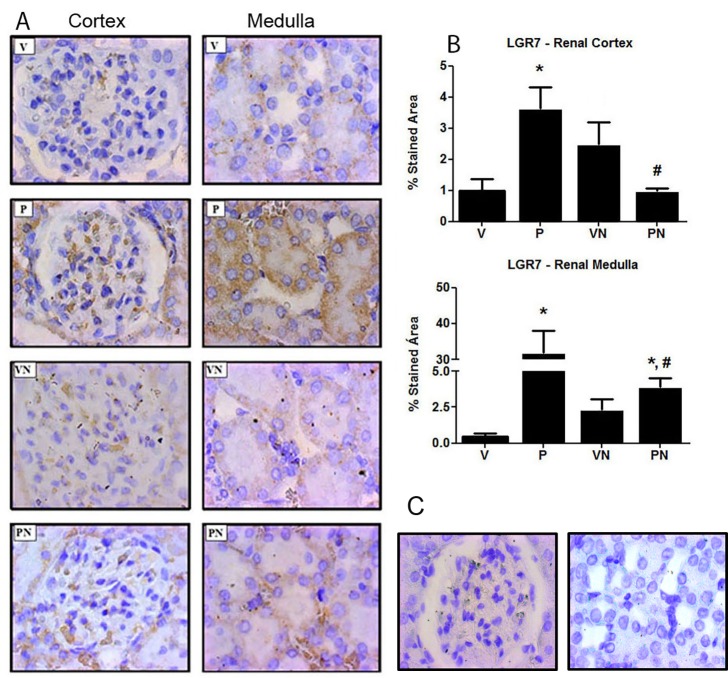
Expression of relaxin receptor (LGR7) (**A**) Representative immunohistochemical analysis showing distribution of LGR7 in renal cortex and medulla (1000× magnification). (**B)** Quantitative analysis of LGR7 staining, n = 4/group. (**C**) Representative slides of negative control, with no primary antibody was omitted.; *p<0.05 and ^#^p<0.05 vs virgin (V) and pregnant (P) groups, respectively.

The main rat relaxin receptor (LGR7) was overexpressed in the kidney cortex and medulla of the control P group animals. Interestingly, while nicotine exposure induced an increase in LGR7 expression in the VN group animals, it drastically reduced it in the PN group rats (Fig 5).

**Fig 6:**
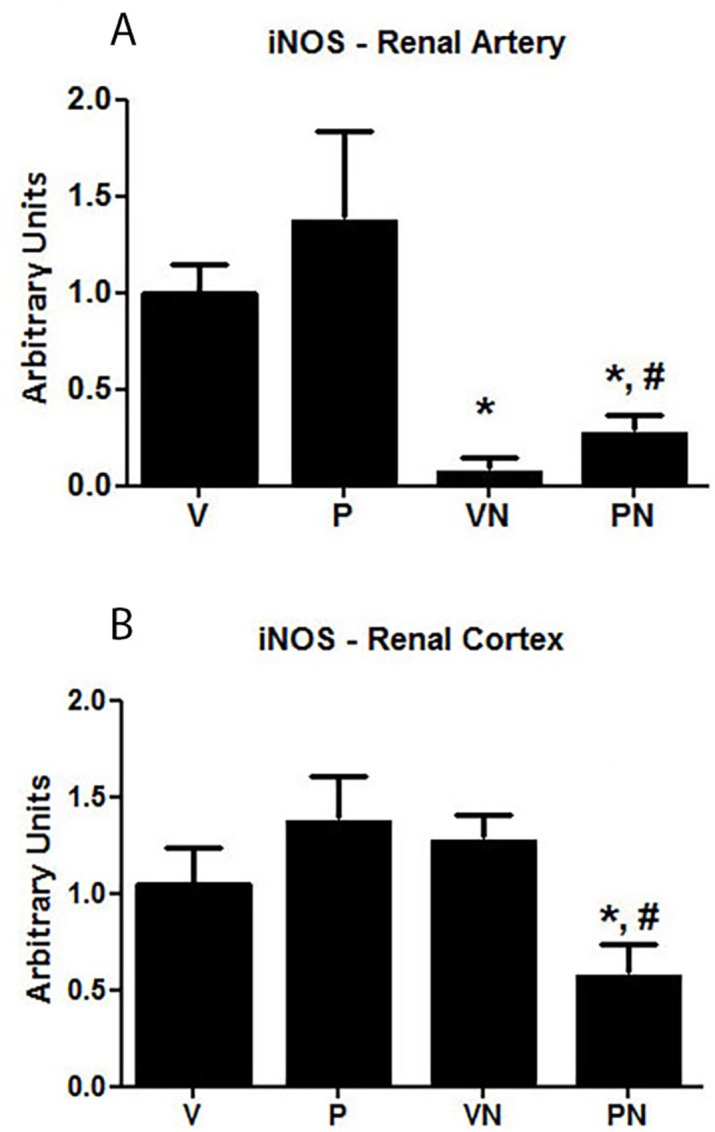
Expression levels of inducible nitric oxide synthase (iNOS) mRNA in (**A**) renal artery and (**B**) renal cortex; n = 6/group, *p<0.05 vs virgin (V) group.

The activity of NO was investigated by determining the expression levels of the iNOS in the kidney and renal artery. Although the iNOS mRNA expression was not changed by pregnancy, nicotine significantly reduced the expression of this isoform in both the cortex and renal artery of the P group rats (Fig 6).

## Discussion

The present study analyzed the systemic and renal changes induced by nicotine in pregnant and virgin rats. Furthermore, we also investigated the influence of pregnancy on the main pathways for the metabolism of nicotine to cotinine via the CYP1A1 enzyme. Similar amounts of cotinine were found in the plasma of both VN and PN group rats exposed to nicotine. However, cotinine concentrations were higher in the urine of pregnant rats than they were in virgin rats, suggesting that there was an increased nicotine metabolism during pregnancy. Interestingly, CYP1A1 was upregulated in the kidney of the control P group rats, suggesting a role for this enzyme in modulating metabolism during normal pregnancy. CYP1A1 is involved in the metabolism of xenobiotics, drugs, and endogenous hormones including those related to pregnancy such as estradiol [34]. In addition, CYPA1 regulates the metabolism of pregnancy-induced vasodilators such as arachidonic acid derivatives [35] and NO [36, 37] that are critical in the regulation of vascular tone and cardiac function. Fatty acids are increased in the maternal circulation [38] and CYP1A1 metabolizes polyunsaturated fatty acids (PUFAs) to vasodilators including NO from omega-3 PUFAs [37] and aracdonic acid from omega-6 PUFA [39]. Tobacco xenobiotics cause various effects on CYP1A1 expression and activity depending on the cell type and the compound. In the present study, we found that nicotine significantly reduced the renal expression of CYP1A1 in the P group, whereas the levels in V group rats were not affected. These results suggest that nicotine may compromise the function of CYP1A1 in maternal adaptation to pregnancy, particularly in the kidney.

As expected, MAP was reduced in the control P group compared to the V group rats, as a sign of the gestational maternal adaptation [1, 40, 41]. The reduction in MAP is caused by systemic vasodilation, which is in turn a consequence of the relative insensitivity of the maternal vascular smooth muscle cells to vasoconstrictor agents including angiotensin II and norepinephrine, together with an increase in the synthesis of vasodilators such as NO and relaxin. In addition, the baroreflex sensitivity was attenuated in pregnant animals, and this behavior has been well described in many species including humans [42]. Consequently, pregnancy may favor the development of orthostatic hypotension and reduce the ability to maintain arterial pressure during hemorrhage [43]. Here, we observed that the pregnancy-induced baroreflex impairment was aggravated by nicotine.

The rSNA was unchanged in the control P group compared with the V group rats. However, analysis of the rSNA showed reduced basal activity in both groups exposed to nicotine compared to their untreated controls possibly as a compensatory mechanism mediated by arterial baroreceptors that control rSNA that apparently do not exhibit short-term resetting[44]. Results obtained from different experimental protocols have shown similar effects of nicotine on MAP in non-pregnant animals [16–18, 45–48] and humans [49, 50]. The hypertensive effects of nicotine have been linked to a direct stimulation of the sympathetic nervous system via excitatory nicotinic receptors, resulting in increased peripheral resistance [14, 46, 47, 51].

Normal pregnancy is characterized by increased blood volume, which is a fundamental requirement for adequate blood supply to the fetus. The expansion of the extracellular volume is primarily determined by renal sodium and water retention mediated by the upregulation of the water channel AQP2 [52] and the main sodium tubular transporters [7]. Indeed, in the present study we observed an upregulation of the NHE_3_ and AQP2 mRNAs, suggesting they play a role in the sodium and water reabsorption in the control P group. The mechanism responsible for this upregulation during pregnancy is not known, but we have previously found that the relaxin receptor, LGR7, was highly expressed in the apical membranes of the proximal tubules (6), suggesting that the action of relaxin may go beyond the intrarenal vasodilation and could interfere with proximal tubular reabsorption (50). Nicotine had no effect on the expression of the tubular transporters in the virgin rats, but, in contrast, it caused a significant reduction in the expression of NHE_3_ and AQP2 in pregnant rats, indicating that nicotine may compromise the tubular capacity to reabsorb sodium and water during pregnancy. Interestingly the downregulation of NHE3 was paralleled by a reduction in the LGR7 expression in the proximal tubule. Taken together, these observations suggest that sodium reabsorption by the proximal tubule in pregnant animals could be at least in part, stimulated by relaxin via LGR7, however, the capacity of relaxin to increase the gene expression of NHE_3_ deserves additional studies.

Altered tubular function and glomerular hyperfiltration are among the hallmark renal physiological changes that characterize a healthy pregnancy in humans[53] and animals [54, 55]. Indeed, the pregnant animals in this study presented with a more elevated GFR than the virgin rats did. Nicotine had no effect on the GFR of the V group; however, it prevented the pregnancy-induced glomerular hyperfiltration. The pregnancy-associated vasodilation, glomerular hyperflow, and hyperfiltration are substantially determined by relaxin [56, 57], which is the vasodilator hormone secreted by the corpus luteum during pregnancy. We observed previously that LGR7 was highly expressed in the tubules and renal arteries of pregnant rats [6]. This finding was confirmed in the present study with the increased LGR7 immunostaining observed in the renal cortex and medulla of the pregnant compared with non-pregnant rats. Furthermore, LGR7 upregulation was severely affected by nicotine exposure, suggesting that an impairment in the relaxin signaling in pregnant animals may be responsible, at least in part, for the reduction in GFR. Therefore, the lower GFR in pregnant rats consuming nicotine constituted another factor that, together with a deficient tubular fluid reabsorption, could compromise maternal adaptation to pregnancy.

NO also has been implicated in the renal vasodilation that occurs during pregnancy because nonselective NOS inhibitors prevent the gestational rise in GFR [58]. Increased NO production in the kidney during pregnancy has been attributed to an upregulation of iNOS and nNOS [59], but recent evidences suggest that nNOS is likely the main isoform responsible to increase NO synthesis in the kidney during pregnancy [60]. In fact, we found iNOS expression unchanged in the renal cortex and renal artery of the control pregnant rats, however iNOS was significantly reduced by nicotine in both virgin and pregnant rats. This result suggests that nicotine may have an impact in the renal NO synthesis independently of gestation, however considering the relevance of NO to maternal adaptation to pregnancy this effect may be worse during pregnancy.

In summary, this study demonstrated that chronic nicotine exposure caused more deleterious effects to pregnant rats than it did to virgin rats. Furthermore, these effects can potentially impair the adaptation of the maternal animal to pregnancy.
